# Therapy of HIV Infection: Current Approaches and Prospects

**Published:** 2016

**Authors:** M. M. Prokofjeva, S. N. Kochetkov, V. S. Prassolov

**Affiliations:** Engelhardt Institute of Molecular Biology, Vavilova Str., 32, Moscow, 119991, Russia

**Keywords:** HIV-1, viral life cycle inhibitors, genome editing, antiviral therapy

## Abstract

The human immunodeficiency virus type 1 (HIV-1) is the causative agent of one of the most dangerous human diseases – the acquired immune deficiency syndrome (AIDS). Over the past 30 years since the discovery of HIV-1, a number of antiviral drugs have been developed to suppress various stages of the HIV-1 life cycle. This approach has enables the suppression of virus replication in the body, which significantly prolongs the life of HIV patients. The main downside of the method is the development of viral resistance to many anti-HIV drugs, which requires the creation of new drugs effective against drug-resistant viral forms. Currently, several fundamentally new approaches to HIV-1 treatment are under development, including the use of neutralizing antibodies, genome editing, and blocking an integrated latent provirus. This review describes a traditional approach involving HIV-1 inhibitors as well as the prospects of other treatment options.

## INTRODUCTION

The development of approaches to the treatment of the HIV infection is one of the most crucial challenges facing biomedical chemistry. The medications used currently are aimed at suppressing one of the key steps of the infection: the initial contact of the virus with the cell, entry, synthesis of the DNA provirus, its transfer into the nucleus and integration into the host cell genome, and the synthesis and maturation of new virions [1]. HIV-1 is highly variable because HIV-1 reverse transcriptase (RT) lacks proofreading exonuclease activity, which results in error-associated transcription. This variability leads to the formation of many mutant viral forms, some of which are drug-resistant [2]. Because drug-resistant viral forms constantly emerge in HIV-infected individuals and are found in so-called primary patients who have undergone no previous treatment with anti-HIV drugs, the search for agents to effectively suppress HIV-1 mutant forms remains topical.

## LIFE CYCLE INHIBITORS

**HIV-1 life cycle**

**Fig. 1:**
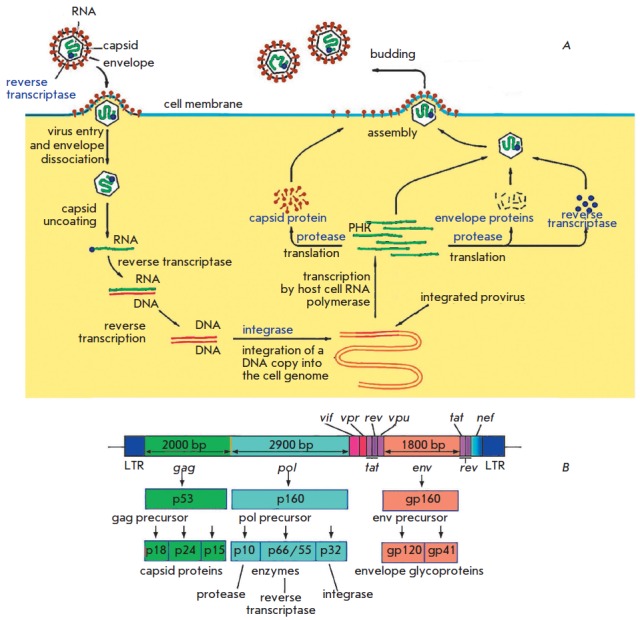
The life cycle (A) and genome structure (B) of HIV-

The life cycle of HIV-1 is schematically depicted in *Fig. 1A*. The initial contact of the virus with an uninfected cell occurs through non-specific binding to the heparan sulfates located on the cell membrane surface. Following this initial contact, viral envelope proteins specifically interact with cell surface proteins (receptors). The receptor for HIV-1 is CD4 (a T cell receptor from the immunoglobulin superfamily) that interacts with the viral envelope glycoproteins gp120 and gp41. HIV-1 uses the chemokine receptors CCR5 and CXCR4 as co-receptors [3]. Mutations in the *CCR5 *gene can significantly affect the infectious process. For example, deletion of 32 bp in the *CCR5 *gene coding region (Δ32 CCR5) results in intracellular synthesis of a CCR5 truncated form that is not exposed on the cell membrane surface. These cells are resistant to HIV-1 strains that use CCR5 as a co-receptor (R5-strains) [4, 5]. The* CXCR4 *gene mutations that induce resistance of the cells to infection are currently unknown.

After fusion of the cell and viral membranes, the capsid enters the cytoplasm and dissociates. This step is followed by reverse transcription, when a DNA copy is synthesized on the viral genomic RNA template, which is accompanied by RNA degradation and synthesis of the second DNA strand. All three steps are implemented by one enzyme, RNA-dependent DNA polymerase, from the viral nucleocapsid. The final product of the polymerase reaction is a double-stranded DNA provirus that contains all viral genes and is flanked by long 3’- and 5’-terminal repeats (LTRs). LTR includes regulatory elements, in particular a promoter and enhancers, which perform important functions in the retrovirus life cycle.

The DNA provirus integrates into the infected cell genome, which is required for the subsequent replication of the viral genome and permanent expression in the infected cells. The integration involves the preintegration complex (PIC) consisting of viral integrase, RT, and a number of cellular proteins [6]. Following this integration, the integrated DNA provirus acts as a part of the host genome, as an independent transcriptional unit. Subsequent transcription of the integrated provirus, as well as processing and splicing of the newly produced viral RNA, is performed by cellular enzymes. The synthesized viral RNA undergoes alternative splicing. The HIV-1 accessory proteins Tat, Rev, Vpu, Vpr, and Vif are translated from a double-spliced RNA (*Fig. 1B*). The regulatory Nef protein and the envelope protein (Env) precursor, which are necessary at later stages of the viral life cycle, are synthesized from the single-spliced RNA. The unspliced viral RNA is incorporated into the capsid of the newly formed viral particles and also serves as a template for the synthesis of the Gag and Gag/Pol precursor proteins encoded by the genes *gag *(structural proteins: matrix MA (p17), capsid CA (p24), and nucleocapsid NC (p7)) and *pol *(viral enzymes: reverse transcriptase (p66/51), integrase (p32), and protease (p10)). Initially, the virus forms as a non-infectious immature virion that buds from the infected cell membrane. After budding, virus maturation occurs when precursor proteins are cleaved by viral protease and the cleavage products start performing their functions in the viral particle [7].

**Reverse transcriptase inhibitors**

**Fig. 2:**
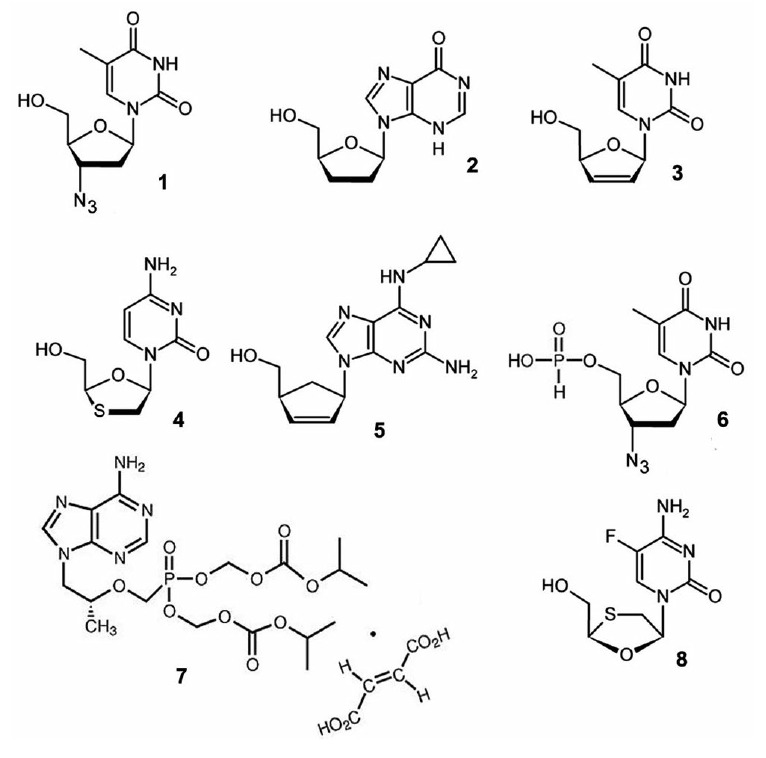
Nucleoside and nucleotide HIV-1 reverse transcriptase inhibitors. The numbering corresponds to that of *Table 1*.

Most of the drugs now used affect a particular HIV-1 enzyme: reverse transcriptase, integrase, or protease (*Table 1*). RT inhibitors may be conventionally divided into two groups: nucleoside and nucleotide reverse transcriptase inhibitors (NRTIs) and non-nucleoside reverse transcriptase inhibitors (NNRTIs). Nucleoside and nucleotide analogues are a group made of the earliest HIV replication inhibitors approved for clinical use [8] (*Fig. 2*). These compounds are enzyme substrate precursors, not an active form of an inhibitor. Upon entering the cell, they are converted (via phosphorylation by cellular kinases) to nucleoside triphosphate analogues that act as substrates in the synthesis of proviral cDNA. Insertion of a NRTI into a growing cDNA chain leads to reverse transcription termination due to the lack of a 3’-hydroxyl group. Therefore, NRTIs block HIV-1 replication at the early step of its life cycle [9-11].

The first inhibitor in this class was azidothymidine (zidovudine) (**1**). This drug was synthesized in 1964 and was tested as an experimental cell cytotoxin for several years. Clinical trials in 1985 demonstrated that the drug inhibits both the infectious and cytopathic properties of HIV-1 [12]. By 2015, the FDA had approved the clinical use of seven drugs. One drug, nikavir (**6**), which was created in the laboratory of Academician A.A. Kraevskiy at the Engelhardt Institute of Molecular Biology, was approved for use in 1999 and has been widely used in Russia and the CIS countries. Each nucleoside analogue specifically competes with a cellular nucleoside: AZT (**1**), nikavir (**6**), and stavudine (d4T) (**3**) compete with dTTP; emtricitabine (FTC) (**8**) and lamivudine (3TC) (**4**) compete with dCTP; didanosine (ddI) (**2**) and tenofovir (TDF) (**7**) compete with dATP; abacavir (ABC) (**5**) competes with dGTP [13-17].

**Table 1:** Anti-HIV drugs approved for use_*_

Russian name	Latin name	Trade name	FDA approval
Nucleoside reverse transcriptase inhibitor (NRTI)			
Zidovudine (1)	Zidovudine (azidothymidine, AZT, ZDV)	Retrovir	19/03/1987
Didanosine (2)	Didanosine (dideoxyinosine, ddI)	09/10/1991
	Delayed-release didanosine, enteric-coated didanosine, ddI EC)	Videx EC	31/10/2000
Stavudine (3)	Stavudine (d4T)	Zerit	24/06/1994
Lamivudine (4)	Lamivudine (3TC)	Epivir	17/11/1995
Abacavir (5)	Abacavir (ABC)	Ziagen	17/12/1998
Phosphazide (6)	Azidothymidine H-phosphonate	Nikavir	05/10/1999**
Tenofovir (7)	Tenofovir disoproxil fumarate (tenofovir DF, TDF)	Viread	26/10/2001
Emtricitabine (8)	Emtricitabine (FTC)	Emtriva	02/07/2003
Non-nucleoside reverse transcriptase inhibitor (NNRTI)			
Nevirapine** (9)	Nevirapine (NVP)	Viramune	21/06/1996
Nevirapine ХR*** (10)	Extended-release nevirapine (NVP XR)	Viramune XR	25/03/2011
Delavirdine (11)	Delavirdine (delavirdine mesylate, DLV)	Rescriptor	04/04/1997
Efavirenz (12)	Efavirenz (EFV)	Sustiva	17/09/1998
Etravirine (13)	Etravirine (ETR)	Intelence	18/01/2008
Rilpivirine (14)	Rilpivirine (RPV)	Edurant	20/05/2011
Protease inhibitor (РI)			
Saquinavir (15)	Saquinavir (SQV)	Invirase	06/12/1995
Ritonavir (16)	Ritonavir (RTV)	Norvir	01/03/1996
Indinavir (17)	Indinavir (IDV)	Crixivan	13/03/1996
Nelfinavir (18)	Nelfinavir (NFV)	Viracept	14/03/1997
Atazanavir (19)	Atazanavir (ATV)	Reyataz	20/06/2003
Fosamprenavir (20)	Fosamprenavir (FOS-APV, FPV)	Lexiva	20/10/2003
Tipranavir (21)	Tipranavir (TPV)	Aptivus	22/06/2005
Darunavir (22)	Darunavir (DRV)	Prezista	23/06/2006
Integrase inhibitor (INI)			
Raltegravir (23)	Raltegravir (RAL)	Isentress	12/10/2007
Dolutegravir (24)	Dolutegravir (DTG)	Tivicay	13/08/2013
Elvitegravir (25)	Elvitegravir (EVG)	Vitekta	24/09/2014
Other			
Enfuvirtide**** (26)	Enfuvirtide (T-20)	Fuzeon	13/03/2003
Maraviroc***** (27)	Maraviroc (MVC)	Selzentry	06/08/2007
Cobicistat****** (28)	Cobicistat, Tybost (COBI)	Tybost	24/09/2014

^*^Consecutive numbers of compounds correspond to their numbers in figures.

^**^Approved for use in the Russian Federation.

^***^Extended-release nevirapine.

^****^Fusion inhibitor.

^*****^Inhibitor of the virus-co-receptor interaction.

^******^A pharmacokinetic enhancer of atazanavir (19) or darunavir (22) action.

Some NRTIs are highly stable in the cell, which enables long-term virus suppression [8].

Unlike nucleoside inhibitors, nucleotide inhibitors are pre-phosphorylated: thereby the latter need one less phosphorylation step after entering the cell. Like nucleoside inhibitors, nucleotide analogues act as terminators of a growing DNA chain. They contain a phosphonate group that cannot be cleaved by cellular hydrolases, which greatly complicates 3’-5’-exonuclease- mediated excision of the nucleotide analogues inserted into a growing DNA chain compared to the excision of nucleoside analogues. The only nucleotide inhibitor used in anti-HIV therapy is tenofovir (**7**) [1].

To design and synthesize new nucleoside and nucleotide analogues is the objective of many researchers that are developing anti-HIV-1 drugs. New nucleoside analogues are needed, because HIV-1 RT undergoes point mutations, conferring drug resistance to the virus. Clinical studies have demonstrated a significant decrease in drug efficacy in HIV-1-infected patients receiving only AZT for six months [18]. There are viral strains fully resistant to AZT and other nucleoside analogues [19-21].

There are two known mechanisms of RT resistance to nucleoside. The first one is associated with a reduced affinity for artificial substrates compared to that for natural substrates. The second mechanism is based on increased phosphorolytic excision of an incorporated chain terminator [22, 23]. HIV-1 RT, even when lacking 3’-exonuclease activity, is capable of catalyzing pyrophosphorolysis, the reverse reaction of polymerization [24].

**Fig. 3:**
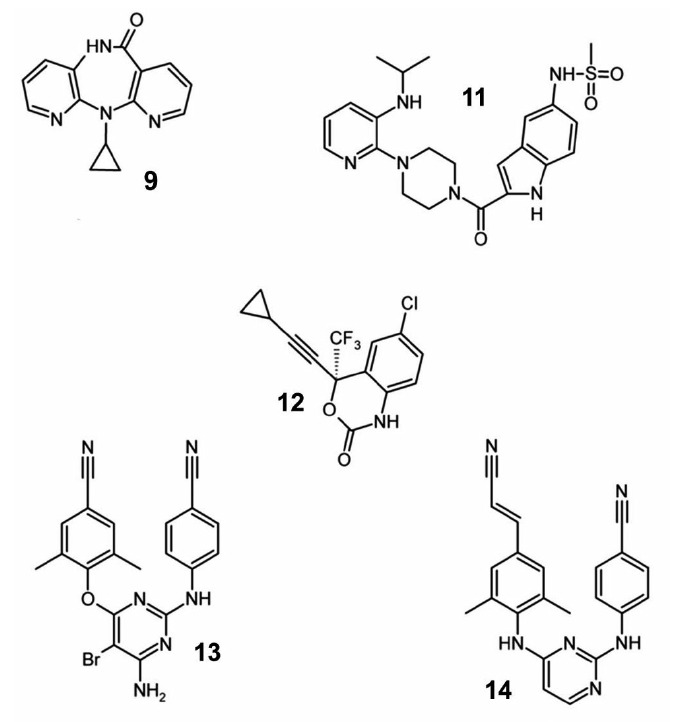
Non-nucleoside HIV-1 reverse transcriptase inhibitors

Non-nucleoside RT inhibitors (*Fig. 3*) are non-competitive inhibitors that bind in the so called hydrophobic pocket near the enzyme’s catalytic site. Because of their hydrophobicity, NNRTIs can enter the cell and do not require any further reactions [25]. Five drugs of this group have been approved for clinical use: nevirapine (**10**), delavirdine (**11**), efavirenz (**12**), etravirine (**13**), and rilpivirine (**14**). The first agent in this group, which was approved as a medication in 1996, was nevirapine [26]. Now, this drug is rarely used, because mutant HIV-1 forms resistant to nevirapine are widespread. Currently, the most commonly used medication in the group, which is prescribed to primary patients, is efavirenz [27].

The chemical structure of NNRTIs is different, but their effect on the enzyme is similar. Inhibitors in this group are specific to HIV-1 RT, but not active against other retroviruses.

Initially, NNRTIs were thought to bind only to an enzyme-substrate complex [28]. Later, NNRTIs were shown to bind to RT regardless of the substrate [29, 30], but some of them have increased affinity to the enzyme in the presence of a substrate [31]. In this case, NNRTIs do not inhibit substrate binding to the active site, but even promote it [32, 33]. This feature enables the application of NNRTIs in combination with NRTIs. Also, NNRTIs were shown to be capable of inhibiting the RNase H activity of RT [34]. ;

Most mutations that confer resistance to NNRTIs occur in the NNRTI binding site. Over 40 mutations conferring *in vivo *and *in vitro *NNRTI resistance in RT have been found. However, if drugs that have been in use for a long time (e.g., nevirapine) are ineffective against a mutant enzyme, new drugs, the so-called second generation NNRTIs (etravirine and rilpivirine), exhibit sufficient inhibitory activity against mutant RT forms [35].

**HIV-1 protease inhibitors**

**Fig. 4:**
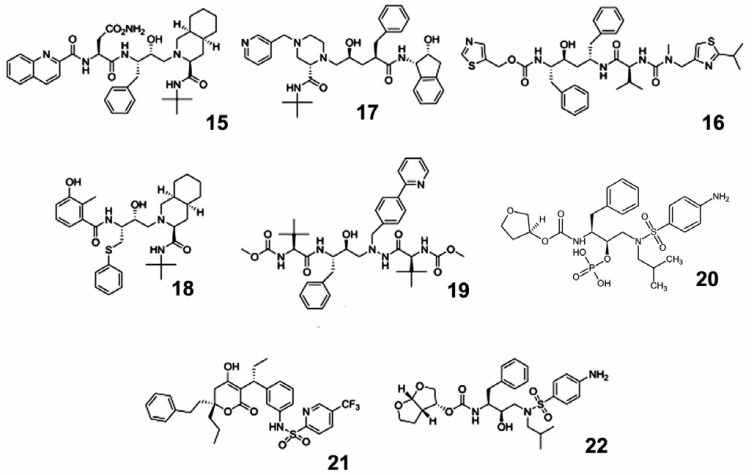
HIV-1 protease inhibitors

A second important group of clinically used inhibitors are protease inhibitors (*Fig. 4*). Most of these compounds are peptidomimetics that act in the same way through binding to the enzyme’s active site. Unlike a natural target, inhibitors are not susceptible to proteolytic cleavage, because they contain hydroxyethylene bonds [–CH_2_–CH(OH)–] instead of peptide bonds [– NH–CO–]. Upon binding to the enzyme’s active center, they compete with natural protease substrates and inhibit the enzymatic activity, which leads to a sharp decrease in the proteolytic processing of viral proteins [36-38]. The first drug in this group of inhibitors was saquinavir (**15**) [39]. Currently, eight protease inhibitors are used; this is the largest group of approved HIV-1 inhibitors (**15–21**). The mechanism inducing HIV-1 resistance to protease inhibitors is based on the replacement of an amino acid residue in the viral protease, which reduces its affinity to an inhibitor, whereas natural substrates continue to interact with the drug-resistant protease [40]. Changing the affinity to natural substrates also reduces the protease efficiency. As a consequence, drug-resistant viral forms are subject to compensatory mutations that reorganize the enzyme’s efficiency and do not directly affect resistance to an inhibitor [41].

**HIV-1 integrase inhibitors**

**Fig. 5:**
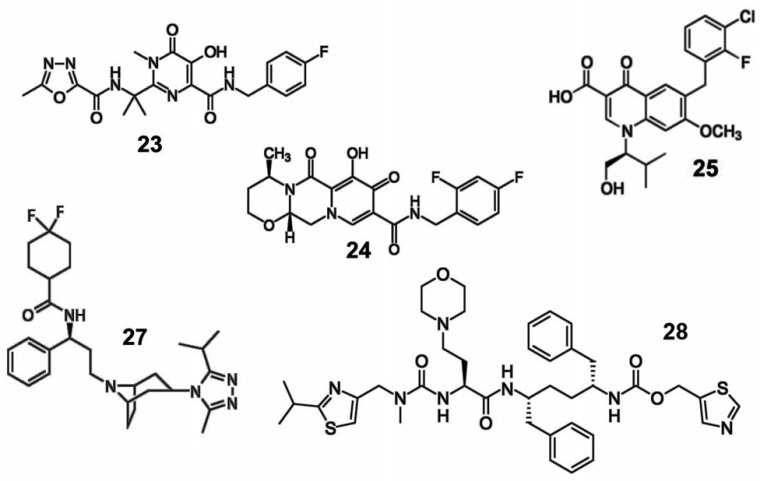
Other inhibitors of the HIV life cycle

Active development of inhibitors in this group began in 2000 when diketone organic acids (e.g., L-731,988) were shown to inhibit the integration and replication of HIV-1 in cell culture in particular, the step of proviral DNA integration into cellular genomic DNA [42]. This was the first indication that integrase inhibitors may be potential antiviral drugs. The first integrase inhibitor, which was approved as a drug in 2007, was raltegravir (isentress) (**23**). Raltegravir exhibited a very high efficiency and quickly became one of the most commonly used drugs [43-45]. Three drugs from this group are now used: raltegravir, dolutegravir (**24**), and elvitegravir (**25**) (*Fig. 5*); they bind to the integration complex and inhibit the integration of proviral DNA into genomic DNA.

**Virus cell entry inhibitors**

Besides inhibitors of HIV-1 enzymes, inhibitors affecting other steps of the viral life cycle have been developed. Virus cell entry inhibitors, which are used in the HIV infection, may be divided into two types: inhibitors of viral and cell membrane fusion and inhibitors of the binding of viral envelope proteins to receptors.

**Fig. 6:**
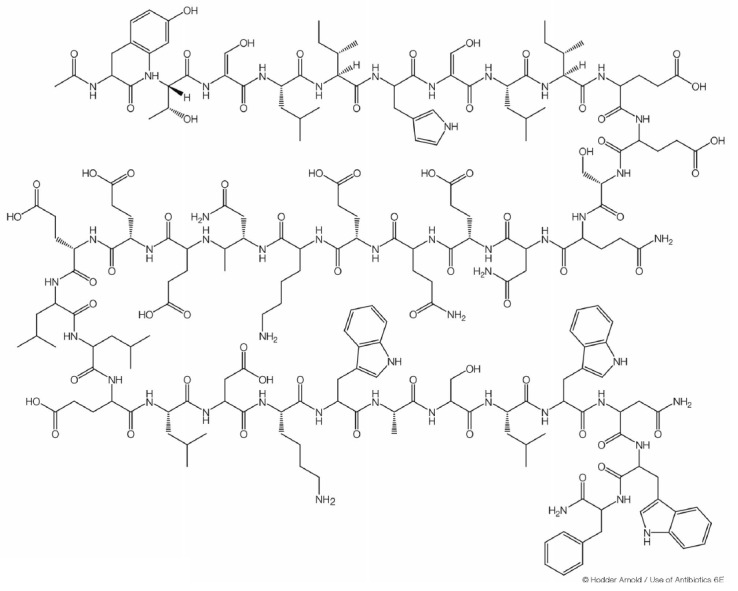
Enfuvirtide structure

At present, only one fusion inhibitor approved as a medication, enfuvirtide (fuzeon) (**26**) (*Fig. 6*), is known. This is a synthetic polypeptide of 36 amino acid residues that mimics a HIV-1 gp41 transmembrane envelope glycoprotein region consisting of heptad repeats, which enables an interaction between enfuvirtide and gp41 [46, 47]. This interaction changes the gp41 conformation, which prevents the fusion of the virus and the cell. Enfuvirtide is the only synthetic polymer among all approved anti-HIV-1 drugs, which explains its high cost. Enfuvirtide is supplied as a solution for injection; it is administered twice a day, making it difficult to use.

Inhibitors of HIV-1 receptor binding need to interact with one of the CCR5 or CXCR4 co-receptors to which a HIV-1 particle binds during cell entry. Currently, this group is represented by the drug maraviroc (selzentry) (**27**) (*Fig. 5*) that interacts with the co-receptor CCR5 [48]. Other inhibitors in this group are under development. The main drawback of CCR5 inhibitors is their inability to affect HIV-1 X4 strains that use the CXCR4 co-receptor [1].

Marine algae polysaccharides and chitosan derivatives are considered as potential antiviral agents. These compounds that act at the virus cell entry step are efficient against HIV-1 and other retroviruses *in vitro*, but they are not approved as drugs, because they do not have a homogeneous composition and a clearly defined structure [49]. Sulfated polysaccharides are structurally similar to heparan sulfates that are primary nonspecific cellular receptors interacting with HIV-1. Presumably, the polysaccharides bind to a HIV-1 envelope protein and prevent its interaction with cell surface receptors. Usually, polysaccharides with a higher molecular weight and a higher degree of sulfation have a more pronounced antiviral activity [50].

Cobicistat (**28**) is another medication approved for clinical use. In contrast to the above-listed compounds, cobicistat is not an inhibitor of a particular step in the HIV-1 life cycle. Cobicistat acts as a pharmacokinetic enhancer of the action of atazanavir or darunavir. It is used as an additive to cocktails used to treat the HIV infection.

**Highly active antiretroviral therapy**

A combination of different inhibitor groups is usual in HIV infection therapy. First, there were nucleoside reverse transcriptase inhibitors combined with non-nucleoside reverse transcriptase inhibitors and protease inhibitors. This method was called highly active antiretroviral therapy (HAART). A combination of three or more inhibitors reduces the dose of each of them, increases the efficiency due to simultaneous action on several steps in the HIV-1 life cycle, and decreases the potential for the emergence of new drug-resistant virus forms. The use of two inhibitor types for a single enzyme, RT, in a cocktail is explained by the fact that they target different functional sites of the enzyme, which underlies enhanced inhibition of the RT function. *Table 2* shows the approved anti-HIV drug cocktails used in HAART.

**Table 2:** Drug combinations (cocktails) used in complex treatment of a HIV infection

Combination	Trade name	FDA approval
Lamivudine/Zidovudine (3TC/ZDV)	Combivir	27/9/1997
Abacavir/Lamivudine/Zidovudine (ABC/3TC/ZDV)	Trizivir	14/11/2000
Abacavir/Lamivudine (ABC/3TC)	Epzicom	2/8/2004
Emtricitabine/Tenofovir (FTC/TDF)	Truvada	2/8/2004
Efavirenz/Emtricitabine/Tenofovir (EFV/FTC/TDF)	Atripla	12/6/2006
Emtricitabine/Rilpivirine/Tenofovir (FTC/RPV/TDF)	Complera	10/8/2011
Elvitegravir/Cobicistat/Emtricitabine/Tenofovir (QUAD, EVG/COBI/FTC/TDF)	Stribild	27/8/2012
Abacavir/Dolutegravir/Lamivudine (ABC/DTG/3TC)	Triumeq	22/8/2014
Atazanavir/Cobicistat (ATV/COBI)	Evotaz	29/1/2015
Darunavir/Cobicistat (DRV/COBI)	Prezcobix	29/1/2015
Elvitegravir/Cobicistat/Emtricitabine/Tenofovir/Alafenamide (EVG/COBI/FTC/TAF)	Genvoya	5/11/2015

## OTHER APPROACHES TO THE TREATMENT OF HIV-1 INFECTION

Over the past 25 years, the attention of researchers has focused primarily on the development and optimization of drugs to suppress HIV-1 replication. The antiviral treatment that is currently used, including HAART, has its limitations. Patients have to take drugs throughout their lives, while new mutant forms of the virus emerge which are resistant to a wide range of drugs. Upon long-term therapy, the drugs may cause a cumulative toxic effect. Many experts agree that a new approach is required to enable the achievement of permanent remission under milder treatment conditions. Also, life cycle inhibitors suppress HIV-1 only in cells with active viral replication, but they do not affect a latent virus. Viral genome copies integrate into the genome of memory T cells (CD4+ T cells) and remain invisible to the immune system [51, 52]. Induction of transcription in these cells leads to the formation of infectious viral particles [53].

The development of an anti-HIV-1 vaccine is considered as an alternative option. The first vaccine was developed in the early 2000s; however, the effectiveness of vaccination was much lower than that of classic anti- HIV drugs [54, 55]. Currently, the activity of so-called broad-spectrum neutralizing antibodies is undergoing clinical trials. The results of preliminary studies suggest that neutralizing antibodies may become promising anti-HIV drugs [56, 57].

Currently, the possibility of affecting a latent virus is being investigated. There are two approaches, called sterilizing and functional cure. The sterilizing cure means complete purging of the body of the viral genome through the destruction of cells bearing the provirus integrated into their genome; the functional cure is a complete suppression of viral activity in the body, which includes blocking latent provirus reactivation.

One of the variants of the sterilizing cure is the transplantation of bone marrow from donors resistant to the HIV infection (e.g., whose genome contains a mutant gene of HIV-1 co-receptors, Δ32 CCR5). As shown in 2009, this approach enabled a complete cure of the HIV infection; i.e., all copies of the viral genome were eliminated from the body. This event was called the “Berlin patient” [58]. The patient underwent radiation therapy and bone marrow transplantation from a donor with Δ32 CCR5. Later, after discontinuation of anti- HIV therapy, the virus could no longer be detected his body. Initially, the case engendered great optimism among physicians. But to date, there have been cases where this approach has not had the desired effect. Therefore, the search for other therapies continues.

**Latent provirus reactivation**

One of the sterilizing cure variants is the “awakening” of latent proviruses. Theoretically, medication that is able to reactivate a latent provirus can successively induce the transcription of the HIV-1 genome, synthesis of viral proteins, and emergence of infectious HIV-1 particles, which would result in the death of the infected cell and decrease the number of latent HIV-1 copies in the human genome. This approach was called “shock and kill.” Cells carrying viral genome copies are supposed either to die due to the cytopathic viral effect or to be destroyed by the immune system. This approach should be combined with maintenance therapy by HIV-1 inhibitors to prevent the spread of the reactivated virus.

Vorinostat, the histone deacetylase inhibitor used in cancer therapy, was studied as a potential anti-HIV-1 drug [59]. As was demonstrated in cells derived from patients and in clinical trials, the inhibitor can induce the transcription of viral genes in some patients. At the same time, vorinostat is cytotoxic and ineffective in all cases, which makes its wide clinical application problematic. Other histone deacetylase inhibitors are undergoing clinical trials [60, 61].

This approach has at least two disadvantages. The first is the potential side effects in the form of non-specific induction of host cell gene transcription. The second is the impossibility to predict whether all the cells harboring induced proviruses die. There is evidence that the immune system cannot recognize all these cells [62]. Progress in this direction hinges on developing a method to effectively destroy cells that harbor the activated provirus.

Along with the investigation of the possibility to “sterilize” the body from all proviral copies, there are studies that endeavor to search for a functional cure that does not require a complete elimination of all copies of the viral genome but effectively inhibits potential viral activity, which excludes the need for a constant use of HIV-1 life cycle inhibitors.

**Inhibition of integrated provirus transcription**

One of the potential therapeutic targets is the HIV-1 Tat protein and the Tat/TAR/P-TEFb complex. Tat is one of the HIV-1 regulatory proteins: a transcription activator. Tat binds to the so-called TAR region of 60 nucleotides located at the 5’-end of a transcribed RNA chain, which does not affect transcription initiation but increases the processivity of RNA polymerase, thereby enhancing transcription many-fold. P-TEFb kinase, the third component of the complex, may also be a target for therapy. Inhibition of the formation and activity of the complex would reduce the transcription level and prevent provirus reactivation [63, 64]. Currently, low-molecular-weight inhibitors affecting either the Tat protein or TAR are under development. Computer simulation is used for the selection of potential low-molecular- weight inhibitors.

The TAR sequence is highly conserved among HIV-1 strains, which makes it possible to select versatile drugs that interact with TAR. Quinolones are effective inhibitors of Tat-dependent transcription [65, 66]. To date, the molecular mechanism of binding to the target has been determined for only a few compounds exhibiting inhibitory activity. For example, 6-aminoquinolone WM5 inhibits the interaction between Tat and TAR through specific binding to the TAR. At the same time, some quinolone derivatives inhibit Tat-dependent transcription, but they do not interact with the TAR/Tat complex [67].

There are a number of low-molecular-weight compounds that interact with the Tat protein and block its binding to TAR. These agents are not yet used for anti- HIV therapy. One of these, the Tat inhibitor triptolide, is undergoing clinical trials. Triptolide is a natural compound isolated from the plant *Tripterygium wilfordii*. Triptolide was demonstrated to promote rapid Tat degradation in cells, thereby inhibiting Tat-dependent transcription [68].

**Genome editing**

A completely new anti-HIV-1 therapy option is gene therapy that includes the editing of the integrated proviral DNA and blocking further functioning of the virus. In 2013, the CRISPR/Cas9 system was used in model HEK293 and HeLa cell lines whose genomes contained an expression cassette comprising a gene encoding GFP and a sequence encoding the HIV-1 Tat protein under the control of the HIV-1 LTR. The CRISPR/ Cas9 system activity for editing the LTR sequence was shown to reduce the GFP expression level in the HEK293 cell line. Similar results were obtained on Jurkat line cells bearing a simulation of latent proviral DNA in their genome, which is an indication of the fact that the CRISPR/Cas9 system may be used to prevent latent provirus reactivation.

It was demonstrated that the TAR sequence can be used as a target for genome editing by the CRISPR/ Cas9 system [69]. Another potential target is the HIV-1 co-receptor CCR5 [70, 71, 72].

However, implementation of this system in clinical practice requires the development of an effective delivery system as well as a series of pre-clinical trials. Definitely, this method is very promising.

## CONCLUSION

The use of HIV-1 inhibitors for antiviral therapy is currently the only method that is actively being applied. In the case of HAART, the use of a combination of drugs aimed at inhibiting different steps of the HIV-1 life cycle minimizes the disadvantages of this approach, because HAART decreases the likelihood of a selection of drug-resistant viral forms and requires smaller doses of all of the drugs, which reduces the potential cumulative toxic effect. New treatment options, which are under development, require further research and clinical trials, but they seem promising for future use.

## Abbreviations

HIV: human immunodeficiency virus
RT: reverse transcriptase
NRTI: nucleoside reverse transcriptase inhibitor
NNRTI: non-nucleoside reverse transcriptase inhibitor
HAART: highly active antiretroviral therapy
LTR: long terminal repeat
